# Acceptability of a Chinese version of volitional help sheet to prevent self-harm repetition: qualitative study

**DOI:** 10.1192/bjo.2023.78

**Published:** 2023-06-23

**Authors:** I-Ting Hwang, Yi-Chun Chen, Christopher J. Armitage, Chia-Yueh Hsu, Shu-Sen Chang

**Affiliations:** Department of Occupational Therapy, College of Medicine, National Cheng Kung University, Taiwan; Institute of Health Behaviors and Community Sciences, College of Public Health, National Taiwan University, Taiwan; Manchester Centre for Health Psychology, School of Health Sciences, University of Manchester, UK; Manchester Academic Health Science Centre, Manchester University NHS Foundation Trust, UK; and NIHR Greater Manchester Patient Safety Translational Research Centre, University of Manchester, UK; Department of Psychiatry, Wan Fang Hospital, Taipei Medical University, Taiwan; Department of Psychiatry, School of Medicine, College of Medicine, Taipei Medical University, Taiwan; and Psychiatric Research Center, Wan Fang Hospital, Taipei Medical University, Taiwan; Institute of Health Behaviors and Community Sciences, College of Public Health, National Taiwan University, Taiwan; Psychiatric Research Center, Wan Fang Hospital, Taipei Medical University, Taiwan; Global Health Program, College of Public Health, National Taiwan University, Taiwan; and Population Health Research Center, National Taiwan University, Taiwan

**Keywords:** Brief intervention, self-harm, suicide, acceptability, patient perspective

## Abstract

**Background:**

Individuals who self-harm have increased suicide rates. Brief interventions are associated with reduced repeated suicide attempts. However, very few previous studies investigated the acceptability of brief interventions before implementing new trials.

**Aims:**

We aimed to explore the perceptions of individuals who self-harm toward a brief intervention, the Chinese version of the volitional help sheet (VHS-C), which encourages people to link a critical situation with an appropriate response.

**Method:**

Fourteen participants who presented to hospitals with self-harm were interviewed about their perspectives regarding the acceptability of the paper- and web-based VHS-C. Data were analysed with the framework method.

**Results:**

The participants could understand the intended goal of the VHS-C by reading the written instructions, but indicated that having verbal instructions would also help. They shared the reasons why they felt the VHS-C was helpful (e.g. relatable contents, useful coping strategies and appropriate instructions that made them feel understood) or unhelpful (e.g., being not specific enough, not useful during the crisis and triggering negative emotional responses). Some indicated that the VHS-C might not be applicable to people experiencing ongoing distress in emergency departments. Most participants preferred the web-based to the paper-based VHS-C, and suggested that the format and frequency of follow-up reminders could leave the patient to decide.

**Conclusions:**

The contents of the VHS-C were acceptable for people who presented to hospitals with self-harm. The VHS-C may be more helpful before individuals encounter suicidal thoughts than when they have an ongoing crisis.

At least 700 000 people worldwide die by suicide every year.^1^ In Taiwan, suicide was the 11th leading cause of death, accounting for 15 deaths per 100 000 people in 2021. Self-harm is a key risk factor for suicide^2^ and could include a wide range of behaviours such as skin cutting, head hitting and self-poisoning.^3^ The suicide rate among individuals with a history of self-harm was 37–49 times greater than that in the general population.^4,5^ Moreover, a recent systematic review showed no decrease in the incidence of repeated self-harm and suicide in those presenting to hospitals with self-harm during the 10 years before the review.^6^ Therefore, there is an urgent need to develop evidence-based interventions to reduce repeated self-harm behaviours.

Acute healthcare settings, such as emergency departments, could be the first contact point for many individuals who self-harm and may provide a window of opportunity for support.^7^ However, existing evidence-based interventions to reduce repeated self-harm behaviours, such as cognitive–behavioural therapy,^8^ are not applicable in acute healthcare settings because of the need for substantial human and time resources. Therefore, a growing body of literature focuses on developing brief interventions, which can be delivered in a single time-limited encounter, require fewer resources, and have greater potential to reach more individuals in need. Recent meta-analyses showed that brief acute-care suicide prevention interventions, such as safety planning and other brief therapeutic interventions, were associated with reduced repeated suicide attempts.^9,10^

## Volitional help sheet

The volitional help sheet (VHS), one type of brief intervention, has been recently examined regarding its effect on reducing repeated suicidal behaviours. The intended goal of the VHS is to apply a self-regulatory strategy known as implementation intention, to support people to link a critical situation (‘if’) with an appropriate response (‘then’), and thus form ‘if–then plans’.^11^ The VHS was developed based on several theories, including the integrated motivational–volitional model of suicidal behaviour^12^ and the transtheoretical model of change.^13^ Two randomised controlled trials investigated the effect of VHS on reducing self-harm and showed inconsistent results. A study in Malaysia found that the VHS could reduce suicidal ideation and behaviour at a 3-month follow-up,^14^ whereas a study in the UK found that the VHS had no overall effect on reducing self-harm repetition at a 6-month follow-up.^15^ One reason for mixed research findings regarding the effectiveness of the VHS could be that the VHS, or part of it, lacked acceptability for some individuals who self-harmed.

It is increasingly acknowledged that acceptability should be a key consideration when developing new interventions. As highlighted by Medical Research Council guidance, developing a deep understanding of the feasibility and acceptability of an intervention is critical to increase the likelihood of successful implementation.^16^ Acceptability is a multifaceted construct, and the perspectives of the target population of the intervention, such as individuals with self-harm, are crucial.^17^ This study aimed to explore the perceptions of individuals presenting to hospitals with self-harm toward the acceptability of the Chinese version of the VHS (VHS-C), with a focus on their perceived intended goal and effectiveness of the VHS-C and feedback on the language clarity, implementation settings and format of the VHS-C.

## Method

### Participants

We used purposeful sampling to recruit participants. The inclusion criteria were that participants were adults (i.e. over 20 years old) with self-harm experiences over the past month. Eligible participants from the in-patient and out-patient units of the Department of Psychiatry at a medical centre in Taipei, Taiwan, were referred to the research team by treating psychiatrists. Patients who were assessed by psychiatrists and found to have hallucination symptoms, current high risk of suicide or limited verbal expression were excluded.

### Data collection and analysis

The original VHS^14^ was forward-translated into Chinese by the research team and then backward-translated into English by a bilingual board-certified psychiatrist. The research team discussed the translation statement by statement to reach a consensus.

**Fig. 1:**
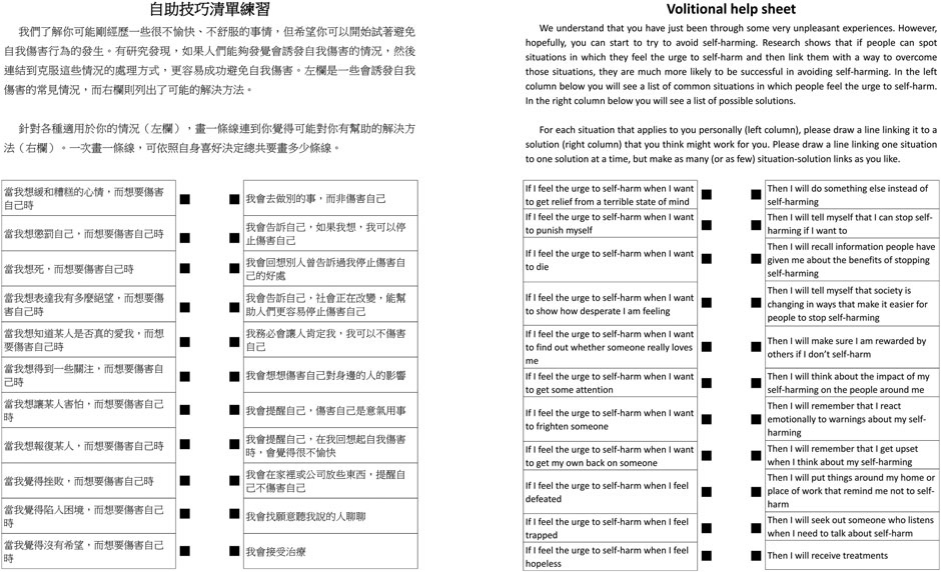
Paper-based Chinese version of the volitional help sheet (VHS-C), with English translation.

**Fig. 2:**
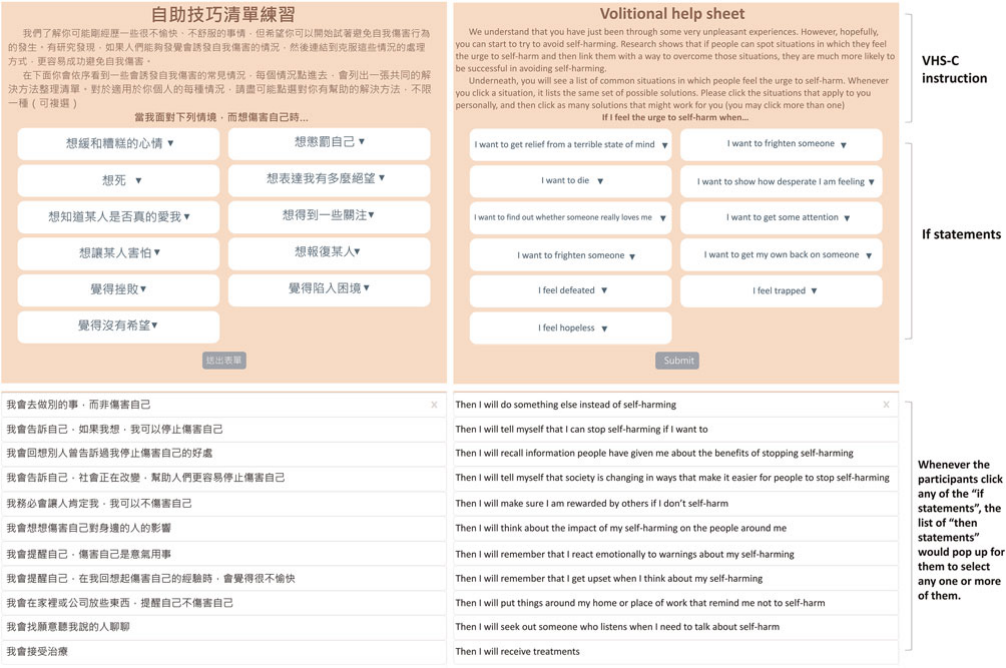
Web-based Chinese version of the volitional help sheet (VHS-C), with English translation.

After providing their written consent, the participants were asked to complete the paper-based VHS-C (Fig. 1) with paper and pencil, and then the web-based VHS-C (Fig. 2) on their own. The web-based VHS-C was designed to be accessible through smartphones or tablets. The participants were provided with a tablet to complete the web-based VHS-C in the study. After completing both formats of the VHS-C, the participants were interviewed for their perspectives on the VHS-C. The interview topics included the following: if they found the VHS-C potentially helpful in reducing future self-harm behaviours, if they experienced any difficulties in understanding the listed critical situations and solutions (i.e. ‘if–then’ statements) in the VHS-C, if they felt that anything missing in the VHS-C and their preferred format of the VHS-C (i.e. paper-based versus web-based). The interviews were conducted between November 2017 and May 2018, lasting 40–60 min each.

The interviews were audio-recorded and analysed using the framework method, with the following steps: transcript, familiarisation with the interview, coding, developing a working analytical framework, applying the analytical framework, charting the data into the framework matrix and interpreting the data.^18^ The recorded interviews were transcribed verbatim. The first and second authors read and re-read each transcript, and proposed potential coding labels independently. Then, the first author incorporated the agreed coding labels to develop the initial analytical framework (i.e. codebook or coding manual). The analytical framework was then revised and finalised based on multiple discussions among the research team members. The first author then applied the final version of the analytical framework to each transcript consistently. We used the qualitative analysis software package ATLAS.ti for Windows (version 8.4.26.0, ATLAS.ti Scientific Software Development GmbH, Berlin, Germany; https://atlasti.com) to summarise the data as a framework matrix and to compare the experiences across participants. The research team members met as a group to identify themes regarding the perceived intended goal and effectiveness of the VHS-C, the clarity of the ‘if–then’ statements and the preferred implementation settings and format of the VHS-C.

The authors assert that all procedures contributing to this work comply with the ethical standards of the relevant national and institutional committees on human experimentation and with the Helsinki Declaration of 1975, as revised in 2008. All procedures involving human patients were approved by the National Taiwan University Hospital Research Ethics Committee (approval number 201708008RINB), and the trial was registered with ClinicalTrials.gov (identifier NCT03376113).

## Results

Fourteen participants completed the interviews. The participants comprised five males and nine females aged 20–47 years. All of the participants reported having a psychiatric diagnosis (Table 1). We categorised participants’ perceptions of the VHS-C into four themes: (a) the perceived intended goal of the VHS-C, (b) the perceived effectiveness of the VHS-C, (c) the clarity of ‘if–then’ statements and suggestions, and (d) the preferred implementation settings and format. Supporting quotes for each theme are provided in Table 2.

**Table 1:** Demographic and self-harm characteristics of the participants

Identifier	Age (years)	Gender	Main psychiatric diagnosis	Self-harm (most recent episode)	
				Time	Method
1	28	Female	Major depressive disorder	1 month ago	Hanging
2	20	Female	Adjustment disorder	2 weeks ago	Overdose
3	56	Female	Major depressive disorder	2 weeks ago	Banging against a wall
4	40	Male	Adjustment disorder	2 weeks ago	Gassing
5	24	Female	Major depressive disorder	3 weeks ago	Wrist cutting
6	44	Female	Schizoaffective disorder	1 week ago	Overdose
7	24	Female	Bipolar disorder type 2	1 month ago	Overdose
8	20	Female	Major depressive disorder	5 months ago	Carbon monoxide poisoning
9	25	Male	Bipolar disorder type 2	1 month ago	Wrist cutting
10	47	Female	Major depressive disorder	2 weeks ago	Charcoal burning
11	31	Female	Major depressive disorder	2 weeks ago	Overdose
12	23	Male	Schizophrenia	2 weeks ago	Stabbing himself in the chest
13	45	Male	Major depressive disorder	8 months ago	Overdose
14	29	Male	Major depressive disorder	1 month ago	Grabbing sharp items

All participants were recruited from an in-patient ward except for participant 14, who was recruited from the out-patient clinic.

**Table 2:** Participant quotes

Key aspects	Quotes from the participants
The perceived intended goal of the VHS-C	**Clear:** ‘I could understand how it [the VHS-C] works. It lists some situations [‘if’ statements] to help you become aware of these situations and provides some actions [‘then’ statements] to avoid this [self-harm] behaviour, so the likelihood of doing such behaviours might decrease. I guess this is what it means to do.’ (participant 9) **Unclear:** ‘To me, it's [VHS-C] like I have to find the answer for each question.’ (participant 3) ‘I feel it's [VHS-C] a questionnaire.’ (participant 13)
The perceived effectiveness of the VHS-C	**Helpful (relatable ‘if’ statements):** ‘I felt these sentences [‘if’ statements] were similar to my experiences.’ (participant 7) **Helpful (new coping strategies):** ‘I felt it [VHS-C] would be helpful, because it provides me with some new approaches that I didn't think of before, and I might try these approaches in the future, such as putting stuff at home or in the office to remind me of not harming myself.’ (participant 9) **Helpful (feelings of being understood and accepted):** ‘I felt I wasn't feeling blamed while reading it [the written instructions of the VHS-C]. I think having this sentence, “We understand that you have just been through some very unpleasant experiences”, is important because the thing I'm afraid of the most is that my pain is not recognised … I felt I was understood while reading it.’ (participant 8) **Unhelpful (not specific enough):** ‘It only says, “I will seek out someone who listens,” but it would have been more helpful if it could list the exact person to talk to.’ (participant 5) **Unhelpful (not useful ‘at the moment’):** ‘While I was thinking about hurting myself, I just wanted to get away from the pain quickly, so I don't think the VHS-C would be helpful for me at the moment.’ (participant 8) **Unhelpful (negative emotional responses):** ‘I don't feel it [VHS-C] helpful. While reading the sentence, “Then I will remember that I react emotionally to warnings about my self-harming”, the first thing coming to my mind was, “I'm emotional”, and then I felt hopeless. Another example, while I was reading this sentence, “Then I will tell myself that I can stop self-harming if I want to”, I felt I was not understood as I couldn't help it … I felt some sentences could be hurtful.’ (participant 2)
The clarity of ‘if–then’ statements and suggestion of new statements	**Statements that lack clarity:** ‘I don't understand this sentence, “Then I will tell myself that society is changing in ways that make it easier for people to stop self-harming”, … does it mean that now we have more medical resources, like more psychiatry departments or more information?’ (participant 9) **Examples of new suggested ‘if–then’ statements:** ‘If I feel the urge to self-harm when I feel betrayed.’ (participant 13) ‘Then I will tell myself that this situation is temporary.’ (participant 2)
The preferred implementation settings and formats	**Settings:** ‘I think it would be very difficult [to read the VHS-C], because they [people who self-harmed] can't really think about other stuff at the moment. Take me as an example. I was pretty agitated while I was at the emergency department.’ (participant 11) **Paper- versus web-based formats:** ‘If I make mistakes on the paper, it would be hard to change.’ (participant 4) ‘It would look very messy on the paper if I draw multiple lines, but the web-based one looks neat.’ (participant 5) **Reminders:** ‘I think the frequency of the reminders could be individualised. For example, some people want daily reminders, while others want monthly reminders. If they can choose the frequency, they might feel more relaxed and less stressed while receiving the reminders.’ (participant 14)

VHS-C: Chinese version of the volitional help sheet.

### The perceived intended goal of the VHS-C

Four participants indicated that they could understand the intended goal of the VHS-C without any problems by simply reading the written instructions. Some other participants indicated that, on top of the written instructions, the interviewer's verbal instructions also helped them to better understand the intended goal of the VHS-C. By contrast, three participants indicated that, by reading the content of the VHS on their own, they could not perceive that the intended goal of the VHS-C was to help people decrease self-harm behaviours; instead, they perceived the VHS-C as simply a list of questions, as one participant said, ‘It [VHS-C] just asks me some questions but does not solve my problems’ (participant 5).

### The perceived effectiveness of the VHS-C

Six participants shared that they found the VHS-C helpful. The main reasons included (a) the ‘if’ statements are relatable to the participants’ own experiences; (b) the ‘then’ statements provided the participants with new coping strategies that they were not aware of, and the participants were willing to try these new strategies; and (c) the instructions of the VHS-C, such as ‘We understand that you have just been through some very unpleasant experiences’, made them feel understood and accepted.

However, some participants shared that the VHS-C might not be helpful because of the following reasons. First, nine participants indicated that some ‘if–then’ statements were not specific enough (e.g. the statements did not specify whom they could talk to when having suicidal thoughts). Second, ten participants indicated that the VHS-C might not be helpful when they were ‘at the moment’ of having suicidal thoughts, as a participant said, ‘I feel it would be hard for me to read these sentences when I want to hurt myself’ (participant 2). Some indicated that they might not check the VHS-C or would be unable to read it at the moment. Finally, three participants reported that the VHS-C might trigger negative emotions, such as feeling frustrated or being condemned. Examples are included in Table 2.

### The clarity of the ‘if–then’ statements and suggestions

The participants indicated that most ‘if–then’ statements were clear, but they found some ‘then’ statements confusing. For example, seven participants indicated that the statement, ‘Then I will make sure I am rewarded by others if I don't self-harm’, was hard to understand. Four participants were confused by the statement, ‘Then I will tell myself that society is changing in ways that make it easier for people to stop self-harming’, as they found it unclear about the specific kinds of societal changes that the statement was referring to, as one participant said, ‘I don't understand this one. I felt the society is getting worse’ (participant 5).

Seven participants suggested adding more ‘if–then’ statements that could reflect their lived experiences. Examples were included in Table 2. One participant suggested that the VHS-C could provide open-ended spaces for them to fill in additional ‘if–then’ statements based on their experiences.

### The preferred implementation settings and format

When asked if emergency departments would be an appropriate setting to implement the VHS-C, half of the participants disagreed. The participants felt that some individuals who have suicidal thoughts or behaviours might not be ready to read through the VHS-C at emergency departments as they may be experiencing emotional outbursts or feeling uncomfortable physically at the moment.

Regarding the comparison between paper-based and web-based versions of the VHS-C, the majority of participants preferred the web-based version of the VHS-C (nine out of 14). The main reason for preferring the web-based version was that, compared with the paper-based version that could have multiple lines on the same page, the web-based version made the VHS-C more visually appealing and easier to use because they could focus on the ‘if–then’ statement one at a time. Regarding the preferred format, texts and emails received a similar number of supporters. The preferred frequency of follow-up reminders ranged from one per week to one every 4 months. The participants indicated that the preference could depend on each individual's habits and personal situations, so they should decide what works best for them.

## Discussion

We conducted interviews with 14 individuals with self-harm experiences and explored their perceptions regarding the acceptability of the VHS-C. The content of the VHS-C was felt to be acceptable, and participants also reported the reasons why the VHS-C could be helpful or unhelpful, suggestions for improvement, that emergency departments may not be an appropriate setting to implement the VHS-C, and their preferred format of the VHS-C, as well as the preferred format and frequency of follow-up reminders.

Although our participants could understand the intended goal of the VHS-C based on the current written instructions, they would like more information and verbal instructions. Similarly, the acceptability studies in the UK highlighted the need for clearer instructions,^19,20^ which would be helpful for people to understand the purpose of the VHS.

We noted two key factors that may influence the acceptability of a brief intervention, like the VHS-C, in individuals with self-harm experiences. The first factor is relevance, i.e. if the target population find the intervention relevant to their lived experiences and needs. In the UK studies, participants also highlighted that the intervention should contain relevant situations, and the relevance level may influence how the participants felt when using the VHS and their confidence in using it.^19,20^ Given the diverse experiences among individuals who self-harm, one possible way to enhance the relevance is to provide open-ended spaces so that individuals could add ‘if–then’ statements based on their own experiences. Another way is to revise the instructions to proactively acknowledge the diversity of individual experiences and that not all statements will be perceived as relevant to everyone, as shown in the UK study.^19^ For example, the instructions could be revised to: ‘The experiences of each person might vary a lot. We try to provide a wide range of situations to capture the experiences as much as possible. You might find some statements relevant or irrelevant to your situation. Please feel free to skip to the next statement if the statement does not fit your situation’.

The second factor is the potential emotional responses triggered by the brief intervention. In our study, feelings toward the VHS-C included a feeling of being understood or judged. In the UK study, some participants pointed out that the VHS may induce negative emotions, such as feeling guilty for wanting to self-harm.^20^ One way to address this concern is to remove or revise the statements and wordings that may lead to negative emotions.

Our participants pointed out two confusing statements. One of them, ‘Then I will make sure I am rewarded by others if I don't self-harm’, was also considered inappropriate in the UK study and removed in the revised version.^20^ The VHS-C was not considered by our participants to be helpful ‘at the moment’ of having suicidal thoughts, and they pointed out that emergency departments might not be the most appropriate place to implement the VHS-C. In the UK, some participants indicated that the VHS might be more useful before the crisis point,^20^ and some participants believed that people might benefit from the VHS only in certain situations or contexts, such as having the willingness to change.^19^ More research is needed to explore when and how potential users want to use the VHS/VHS-C to support themselves.

### Strengths and limitations

This is among the first studies exploring the acceptability of the VHS-C and ways of improvement from the perspectives of individuals with self-harm experiences. According to the findings, we revised the VHS-C. The web-based VHS-C can be found in Supplementary Fig. 1 available at https://doi.org10.1192/bjo.2023.78/. However, there are several limitations of this study. The findings were restricted to the experiences of 14 participants with mental health diagnoses recruited from the in-patient and out-patient units. This group received more intensive care than those who did not seek medical help. Many individuals with self-harm experiences did not seek mental health services,^21^ and their perspectives regarding the VHS-C could be different. Further research is needed to include a more diverse group of participants to inform the design and implementation of aftercare interventions that use the VHS-C.

## Data Availability

Because of the nature of this research, participants of this study did not agree to share their data.

## Supplementary material

For supplementary material accompanying this paper visit http://doi.org/10.1192/bjo.2023.78. click here to view supplementary material

## References

[1] World Health Organization (WHO). *Suicide Worldwide in 2019*. WHO, 2021 (https://www.who.int/publications/i/item/9789240026643).

[2] Favril L, Yu R, Uyar A, Sharpe M, Fazel S. Risk factors for suicide in adults: systematic review and meta-analysis of psychological autopsy studies. BMJ Ment Health 2022; 25(4): 148–55. 10.1136/ebmental-2022-300549 PMC9685708 36162975

[3] Skegg K. Self-harm. Lancet 2005; 366(9495): 1471–83. 16243093 10.1016/S0140-6736(05)67600-3

[4] Olfson M, Wall M, Wang S, Crystal S, Gerhard T, Blanco C. Suicide following deliberate self-harm. Am J Psychiatry 2017; 174(8): 765–74. 28320225 10.1176/appi.ajp.2017.16111288

[5] Hawton K, Bergen H, Cooper J, Turnbull P, Waters K, Ness J, et al. Suicide following self-harm: findings from the multicentre study of self-harm in England, 2000–2012. J Affect Disord 2015; 175: 147–51. 25617686 10.1016/j.jad.2014.12.062

[6] Carroll R, Metcalfe C, Gunnell D. Hospital presenting self-harm and risk of fatal and non-fatal repetition: systematic review and meta-analysis. PLoS One 2014; 9(2): e89944. 24587141 10.1371/journal.pone.0089944 PMC3938547

[7] Betz ME, Wintersteen M, Boudreaux ED, Brown G, Capoccia L, Currier G, et al. Reducing suicide risk: challenges and opportunities in the emergency department. Ann Emerg Med 2016; 68(6): 758–65. 27451339 10.1016/j.annemergmed.2016.05.030

[8] Hawton K, Witt KG, Salisbury TLT, Arensman E, Gunnell D, Hazell P, et al. Psychosocial interventions following self-harm in adults: a systematic review and meta-analysis. Lancet Psychiatry 2016; 3(8): 740–50. 27422028 10.1016/S2215-0366(16)30070-0

[9] Doupnik SK, Rudd B, Schmutte T, Worsley D, Bowden CF, McCarthy E, et al. Association of suicide prevention interventions with subsequent suicide attempts, linkage to follow-up care, and depression symptoms for acute care settings: a systematic review and meta-analysis. JAMA Psychiatry 2020; 77(10): 1021–30. 32584936 10.1001/jamapsychiatry.2020.1586 PMC7301305

[10] Nuij C, van Ballegooijen W, De Beurs D, Juniar D, Erlangsen A, Portzky G, et al. Safety planning-type interventions for suicide prevention: meta-analysis. Br J Psychiatry 2021; 219(2): 419–26. 35048835 10.1192/bjp.2021.50

[11] Gollwitzer PM, Sheeran P. Implementation intentions and goal achievement: a meta-analysis of effects and processes. Adv Exp Soc Psychol 2006; 38: 69–119.

[12] O'Connor RC, Kirtley OJ. The integrated motivational-volitional model of suicidal behaviour. Philos Trans R Soc Lond B Biol Sci 2018; 373(1754): 20170268. 30012735 10.1098/rstb.2017.0268 PMC6053985

[13] Prochaska JO, DiClemente CC. Stages and processes of self-change of smoking: toward an integrative model of change. J Consult Clin Psychol 1983; 51(3): 390. 6863699 10.1037//0022-006x.51.3.390

[14] Armitage CJ, Rahim WA, Rowe R, O'Connor RC. An exploratory randomised trial of a simple, brief psychological intervention to reduce subsequent suicidal ideation and behaviour in patients admitted to hospital for self-harm. Br J Psychiatry 2016; 208(5): 470–6. 26743808 10.1192/bjp.bp.114.162495

[15] O'Connor RC, Ferguson E, Scott F, Smyth R, McDaid D, Park AL, et al. A brief psychological intervention to reduce repetition of self-harm in patients admitted to hospital following a suicide attempt: a randomised controlled trial. Lancet Psychiatry 2017; 4(6): 451–60. 28434871 10.1016/S2215-0366(17)30129-3 PMC5447136

[16] Skivington K, Matthews L, Simpson SA, Craig P, Baird J, Blazeby JM, et al. A new framework for developing and evaluating complex interventions: update of medical research council guidance. BMJ 2021; 374: n2061. 10.1136/bmj.n2061 PMC8482308 34593508

[17] Sekhon M, Cartwright M, Francis JJ. Acceptability of healthcare interventions: an overview of reviews and development of a theoretical framework. BMC Health Serv Res 2017; 17(1): 88. 28126032 10.1186/s12913-017-2031-8 PMC5267473

[18] Gale NK, Heath G, Cameron E, Rashid S, Redwood S. Using the framework method for the analysis of qualitative data in multi-disciplinary health research. BMC Med Res Methodol 2013; 13: 117. 24047204 10.1186/1471-2288-13-117 PMC3848812

[19] Keyworth C, Quinlivan L, Leather JZ, Armitage CJ. Exploring the acceptability of a brief online theory-based intervention to prevent and reduce self-harm: a theoretically framed qualitative study. BJPsych Open 2022; 8(6): e184. 36221254 10.1192/bjo.2022.568 PMC9634605

[20] Keyworth C, O'Connor R, Quinlivan L, Armitage CJ. Acceptability of a brief web-based theory-based intervention to prevent and reduce self-harm: mixed methods evaluation. J Med Internet Res 2021; 23(9): e28349. 34518153 10.2196/28349 PMC8479604

[21] Han J, Batterham PJ, Calear AL, Randall R. Factors influencing professional help-seeking for suicidality: a systematic review. Crisis 2018; 39(3): 175. 29052431 10.1027/0227-5910/a000485

