# Supplementary material for: Acceptability of a Chinese version of volitional help sheet to prevent self-harm repetition: qualitative study
Source: BJPsych Open. 2023 Jun 23;9(4):e115. doi: 10.1192/bjo.2023.78 (PMC10304852; doi:10.1192/bjo.2023.78)
Supplement: Supplementary file 1 [file S2056472423000789sup001.docx]

# Supplementary Figure 1. Revised web-based Chinese version of the volitional help sheet (VHS-C) with English translation


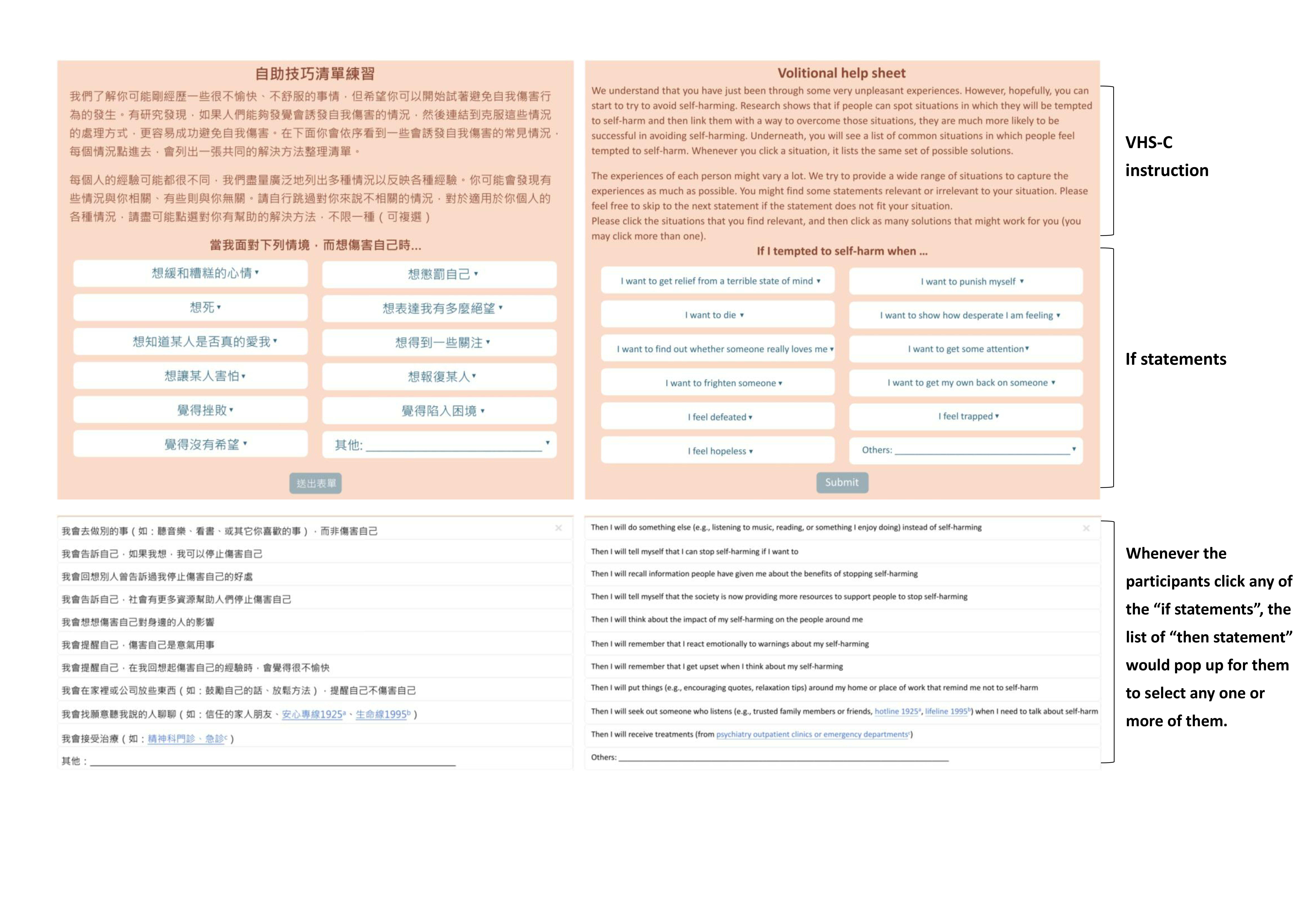


^a^The users can make phone calls to Taiwan’s national suicide prevention hotline (number: 1925) through the link.

^b^The users can make phone calls to Taiwan Lifeline (number: 1995) through the link.

^c^The users can see the Google Maps of the psychiatric outpatient clinics and emergency departments nearby through the link.
